# Peroxisomal ATP Uptake Is Provided by Two Adenine Nucleotide Transporters and the ABCD Transporters

**DOI:** 10.3389/fcell.2021.788921

**Published:** 2022-01-19

**Authors:** Carlo W. T. van Roermund, Lodewijk IJlst, Nicole Linka, Ronald J. A. Wanders, Hans R. Waterham

**Affiliations:** ^1^ Laboratory Genetic Metabolic Diseases, Amsterdam Gastroenterology, Endocrinology and Metabolism, Amsterdam University Medical Centers—Location AMC, University of Amsterdam, Amsterdam, Netherlands; ^2^ Department of Plant Biochemistry, Heinrich-Heine University Düsseldorf, Düsseldorf, Germany

**Keywords:** peroxisome, ABCD, beta oxidation, SLC25A, ATP uptake

## Abstract

Peroxisomes are essential organelles involved in various metabolic processes, including fatty acid β-oxidation. Their metabolic functions require a controlled exchange of metabolites and co-factors, including ATP, across the peroxisomal membrane. We investigated which proteins are involved in the peroxisomal uptake of ATP in the yeast *Saccharomyces cerevisiae*. Using wild-type and targeted deletion strains, we measured ATP-dependent peroxisomal octanoate β-oxidation, intra-peroxisomal ATP levels employing peroxisome-targeted ATP-sensing reporter proteins, and ATP uptake in proteoliposomes prepared from purified peroxisomes. We show that intra-peroxisomal ATP levels are maintained by different peroxisomal membrane proteins each with different modes of action: 1) the previously reported Ant1p protein, which catalyzes the exchange of ATP for AMP or ADP, 2) the ABC transporter protein complex Pxa1p/Pxa2p, which mediates both uni-directional acyl-CoA and ATP uptake, and 3) the mitochondrial Aac2p protein, which catalyzes ATP/ADP exchange and has a dual localization in both mitochondria and peroxisomes. Our results provide compelling evidence for a complementary system for the uptake of ATP in peroxisomes.

## Introduction

Peroxisomes are single-membrane bounded organelles found in cells of all eukaryotic species. They can be involved in a large variety of metabolic pathways which may differ per species but always includes the degradation of fatty acids through β-oxidation. In mammals, including humans, peroxisomes also play an important role in ether phospholipid biosynthesis, fatty acid alpha-oxidation, bile acid synthesis, glyoxylate detoxification and H_2_O_2_ degradation (Wanders and Waterham, 2006; Van Veldhoven, 2010). Genetic defects in the biogenesis and/or functioning of peroxisomes affect these metabolic pathways and usually have severe clinical consequences, as is demonstrated in the Zellweger spectrum disorders (Waterham et al., 2016) and the various single peroxisomal enzyme deficiencies (Wanders, 2018).

In most metabolic pathways, peroxisomes only catalyze a specific subset of enzyme reactions with other reactions catalyzed in the cytosol, mitochondria and/or endoplasmic reticulum (Wanders, 2014). The involvement of different cellular compartments implies that the various metabolites involved, i.e. substrates and products, and co-factors, i.e. NAD, ATP, CoA, need to be transported across the peroxisomal membrane. In the past decades, the enzymology and biochemical functions of peroxisomes have largely been resolved. However, the mechanisms involved in peroxisomal metabolite transport have remained largely unknown. Yet, the importance of this transport is underlined by the existence of two inherited human diseases that are caused by defects in the peroxisomal half ABC transporter proteins ABCD1 and ABCD3 (Wanders, 2018), which function in the peroxisomal import of the CoA esters of very-long-chain fatty acids and branched-chain fatty acids, respectively.

Current consensus holds that peroxisomes are equipped with two fundamentally different mechanisms for metabolite transport across their membrane, which includes 1) diffusion of small Mw metabolites (<400 Da) via channel-forming membrane proteins, and 2) carrier-mediated transport of higher Mw metabolites, such as acyl-CoAs and ATP. Genetic complementation approaches, sequence similarity searches, and proteomic analyses of highly purified peroxisomes of mouse (Mi et al., 2007; Wiese et al., 2007), human (Gronemeyer et al., 2013), plants (Plett et al., 2020), and the yeast *Saccharomyces cerevisiae* (Yi et al., 2002; Chen and Williams, 2018) have led to the identification of several integral peroxisomal membrane proteins which, based on (partial) sequence similarity shared with known transport proteins, may function as peroxisomal metabolite transport proteins.

In mammals, including humans, three half ABC transporter proteins have been identified in the peroxisomal membrane: ABCD1 (also known as adrenoleukodystrophy protein, ALDP) (Aubourg et al., 1993; Mosser et al., 1993), ABCD2 (also known as adrenoleukodystrophy-related protein, ALDRP) (Lombard-Platet et al., 1996; Holzinger et al., 1997) and ABCD3 (also known as 70-kDa peroxisomal membrane protein, PMP70) (Kamijo et al., 1993). These proteins were shown to function as homodimers and import long, very-long-chain and branched-chain acyl-CoA esters into peroxisomes (van Roermund et al., 2012; Okamoto et al., 2018). So far, only three additional mammalian peroxisomal membrane proteins with a presumed function in metabolite transport have been identified. The first one is SLC25A17, also known as PMP34, which, based on sequence similarity, is a member of the mitochondrial carrier family (MCF). Reconstitution experiments in proteoliposomes followed by substrate exchange studies revealed that, *in vitro*, this protein is able to transport CoA, FAD, FMN, and AMP, and to a lesser extent NAD^+^, PAP (adenosine 3′,5′-diphosphate) and ADP (Agrimi et al., 2012).

The second protein is PXMP2, which was shown to have channel-forming properties (Rokka et al., 2009). The third protein is PXMP4, which shares some similarity with bacterial permeases, but has not been functionally studied (Reguenga et al., 1999; Visser et al., 2007).

Peroxisomes in *S. cerevisiae* contain two half ABC transporters, Pxa1p and Pxa2p, which are involved in the import of long-chain acyl-CoA esters (e.g., C18:1) (Hettema et al., 1996; Shani et al., 1996; Swartzman et al., 1996; Roermund et al., 2008). In contrast to their human orthologues, Pxa1p and Pxa2p were shown to function as heterodimers. Two additional peroxisomal membrane proteins with a presumed function in metabolite transport have been identified in *S. cerevisiae*.

Ant1p is an MCF member with strong similarity to human PMP34 but, in contrast to PMP34, demonstrated to catalyze the exchange of cytosolic ATP for peroxisomal AMP or ADP. AMP is generated upon the intra-peroxisomal ATP-dependent activation of fatty acids by the acyl-CoA synthetase Faa2p (Palmieri et al., 2001; van Roermund et al., 2001) while ADP is generated by the peroxisomal nudix family members NPY1 and PCD1 (Plett et al., 2020).

The second protein, Pex11p, is known to be involved in peroxisomal fission, but also was reported to have transport or channel-forming properties (van Roermund et al., 2000; Mindthoff et al., 2016).

We use *S. cerevisiae* as model system (van Roermund et al., 2003) to unravel the mechanism of metabolite transport across the peroxisomal membrane. In contrast to human cells, in which both mitochondria and peroxisomes perform β-oxidation, fatty acid degradation in yeast cells takes place exclusively in peroxisomes and thus requires the import of fatty acids, and the co-factors ATP and CoA. Medium-chain fatty acids with carbon lengths of 8–12 enter yeast peroxisomes in their free acid form and are activated into CoA esters inside peroxisomes via the peroxisomal acyl-CoA synthetase Faa2p (Hettema et al., 1996; Roermund et al., 2008). This activation is ATP- and CoA-dependent. Long-chain fatty acids, however, are first activated outside peroxisomes and then imported as acyl-CoA ester by the ABC transporter protein complex Pxa1p/Pxa2p (Hettema et al., 1996; Roermund et al., 2008), followed by release of coenzyme A at the luminal side of peroxisomes and re-esterification by a peroxisomal synthetase (van Roermund et al., 2012; Carrier et al., 2019; van Roermund CWT et al., 2020).

Although in yeast, intra-peroxisomal ATP is essential for the peroxisomal β-oxidation of fatty acids following their import via the free fatty-acid route as well as the ABC transporter protein-mediated pathway, relatively little is known about the peroxisomal uptake of ATP except for the above mentioned involvement of Ant1p (Palmieri et al., 2001; van Roermund et al., 2001). Our observation that a knock-out of Ant1p in *S. cerevisiae* does not completely abolishes peroxisomal β-oxidation, however, implied the existence of additional ways to import ATP into peroxisomes. In this study we show that the uptake of ATP into peroxisomes is indeed mediated by different peroxisomal membrane proteins. In addition to Ant1p, these include the ABC transporter protein complex Pxa1p/Pxa2p, which thus catalyzes peroxisomal ATP uptake as well as acyl-CoA import, and the MCF carrier Aac2p, a predominantly mitochondrial protein, which we found partially localized to peroxisomes and which catalyzes the exchange of cytosolic ATP for peroxisomal ADP.

## Results

### Ant1p and the ABC Transporter Protein Complex Pxa1p/Pxa2p Transport ATP Across the Peroxisomal Membrane

**FIGURE 1:**
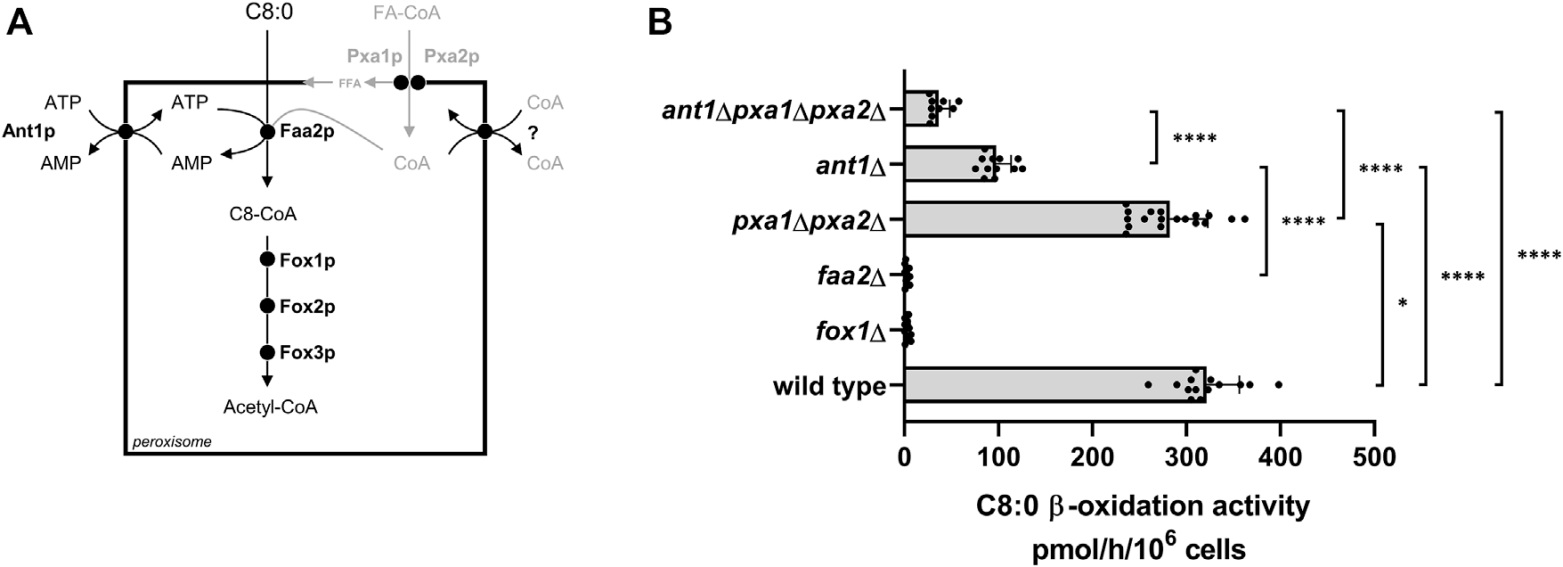
C8:0 β-oxidation activity in wild-type and mutant yeast strains. **(A)** In yeast, β-oxidation of fatty acids occurs exclusively in peroxisomes requiring the import of fatty acids, ATP and CoA. Medium-chain fatty acids (C8–C12) enter yeast peroxisomes as free fatty acids and are subsequently activated to their corresponding CoA ester by the peroxisomal enzyme acyl-CoA synthetase Faa2p. This activation step is ATP and CoA dependent. For completeness, the acyl-CoA uptake route, not involved in the uptake of medium-chain fatty acids, and a yet to identify CoA transporter are depicted in gray. **(B)** Yeast cells were cultured overnight in oleate medium and β-oxidation rates were measured using [1–^14^C] labelled octanoate (C8:0) as substrate. Data are means ± SD of values from 5–18 independent experiments. Only most relevant statistic relations are indicated in Figure 1B, all are given in Supplementary Table S1. ****, ***, **, and * indicate significance with a *p*-value of *p* < 0.0001, *p* < 0.001, *p* < 0.01, and *p* < 0.05 respectively.

We previously showed that in the yeast *S. cerevisiae*, medium chain fatty acids such as octanoate (C8:0) are imported into peroxisomes as free fatty acids. To become substrate for β-oxidation they subsequently are activated into their corresponding fatty acyl-CoA ester by the intra-peroxisomal ATP-dependent acyl-CoA synthetase Faa2 (Figure 1A) (Hettema et al., 1996; Roermund et al., 2008). In accordance with this, the β-oxidation of C8:0 in mutant cells in which the *FAA2* gene is deleted (*faa2Δ*) is fully deficient, similar as in *fox1Δ* cells in which the *FOX1* gene encoding acyl-CoA oxidase, the first enzyme of the β-oxidation pathway, is deleted (Figure 1B). Earlier work also showed that the peroxisomal membrane protein Ant1p functions as an antiporter of ATP against AMP or ADP and thus most probably is responsible for the peroxisomal uptake of ATP required for the intra-peroxisomal activation of fatty acids (Palmieri et al., 2001; van Roermund et al., 2001). Indeed, deletion of *ANT1* resulted in a significant decrease in the C8:0 β-oxidation activity (Figure 1B). However, the C8:0 β-oxidation activity in the *ant1*Δ cells was still ∼30% of the activity measured in wild-type cells, which implied the involvement of additional ATP uptake system(s) in the peroxisomal membrane. To identify these, we measured C8:0 β-oxidation activities in *ant1*Δ cells in which, in addition, genes encoding other known peroxisomal membrane proteins were deleted. Surprisingly, we observed that C8:0 β-oxidation activity was further reduced to ∼10% when we also deleted both *PXA1* and *PXA2* in the *ant1*Δ cells (Figure 1B). In *pxa1*Δ *pxa2*Δ double mutant cells, the C8:0 β-oxidation was only slightly decreased compared to wild-type cells. These findings pointed to a novel, unanticipated role for the ABC transporter protein complex Pxa1p/Pxa2p in peroxisomal ATP uptake in addition to its established role in the peroxisomal import of fatty acyl-CoAs.

### ATP Uptake in Proteoliposomes Prepared From Peroxisomes of Different Mutant Strains

To provide additional evidence for a role of Pxa1p/Pxa2p in peroxisomal ATP uptake, we next studied the uptake of radio-labeled ATP in proteoliposomes prepared from peroxisomal membranes isolated from wild-type cells, *ant1*Δ, *pxa1*Δ *pxa2*Δ, and *ant1*Δ *pxa1*Δ *pxa2*Δ mutant cells.

**FIGURE 2:**
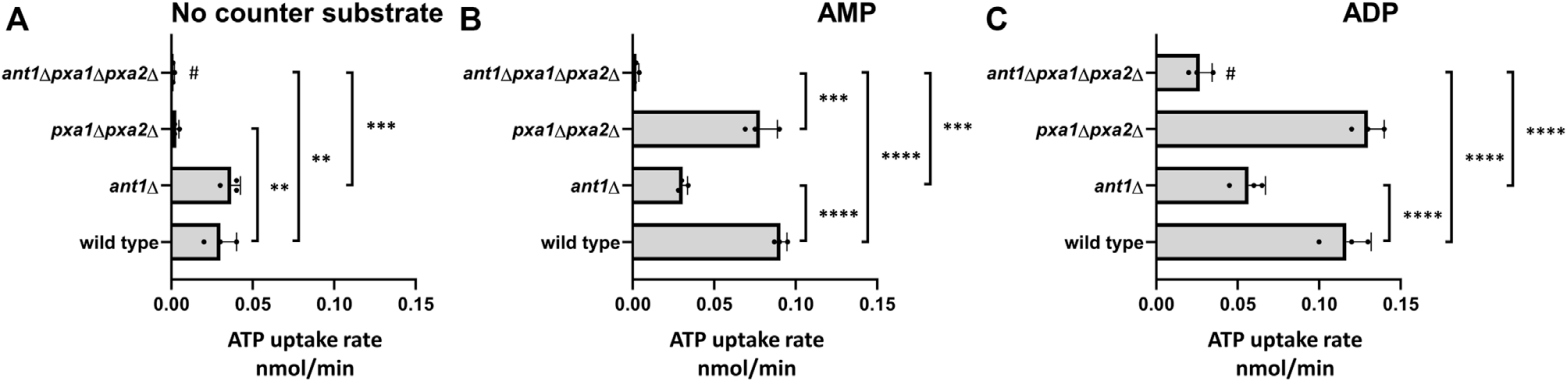
ATP uptake into liposomes reconstituted with peroxisomal membranes purified from different yeast strains. Isolated peroxisomal membranes were reconstituted in proteoliposomes with no counter substrate **(A)**, or pre-loaded with AMP **(B)** or ADP **(C)**. Uptake of [α-^32^P]ATP was measured as described in Methods. Data are means ± SD of values from three independent experiments. Only most relevant statistic relations are indicated in Figure 2, all are given in Supplementary Table S1. ****, ***, **, and * indicate significance with a *p*-value of *p* < 0.0001, *p* < 0.001, *p* < 0.01, and *p* < 0.05 respectively and # indicates significant difference between ADP or no counter substrate.

In the absence of internal adenine nucleotides as counter-exchange substrate, we observed low and similar levels of ATP uptake in proteoliposomes prepared from *ant1*Δ and wild-type cells, while no ATP uptake was observed in proteoliposomes prepared from *pxa1*Δ *pxa2*Δ and *ant1*Δ *pxa1*Δ *pxa2*Δ mutant cells (Figure 2A). When we preloaded the proteoliposomes with AMP, ATP uptake was reduced to ∼30% in proteoliposomes prepared from *ant1*Δ mutant cells, similar in proteoliposomes prepared from *pxa1*Δ *pxa2*Δ mutant cells, and virtually absent in proteoliposomes prepared from *ant1*Δ *pxa1*Δ *pxa2*Δ mutant cells when compared to ATP uptake in proteoliposomes prepared from wild-type cells (Figure 2B). Combined, these findings 1) show that Ant1p is responsible for most of the peroxisomal ATP uptake, 2) confirm that Ant1p functions as peroxisomal antiporter of ATP against AMP (Palmieri et al., 2001; van Roermund et al., 2001); 3) are in agreement with the C8:0 β-oxidation activities measured in the corresponding mutant cells; and 4) support a role for Pxa1p/Pxa2p in unidirectional peroxisomal uptake of ATP.

When we preloaded the proteoliposomes with ADP, ATP uptake was similar in proteoliposomes prepared from *pxa1*Δ *pxa2*Δ mutant cells and wild-type cells, and reduced to ∼45% in proteoliposomes prepared from *ant1Δ* mutant cells. This confirms that Ant1p can also function as an antiporter of ATP against ADP as previously shown (Palmieri et al., 2006). Interestingly, *ant1*Δ *pxa1*Δ *pxa2*Δ mutant cells still showed ∼20% ATP uptake when compared to wild-type cells (Figure 2C), which indicated the involvement of at least one additional peroxisomal ATP transporter that can mediate the exchange of ATP for ADP.

### Direct Measurement of Intra-Peroxisomal ATP Levels

**FIGURE 3:**
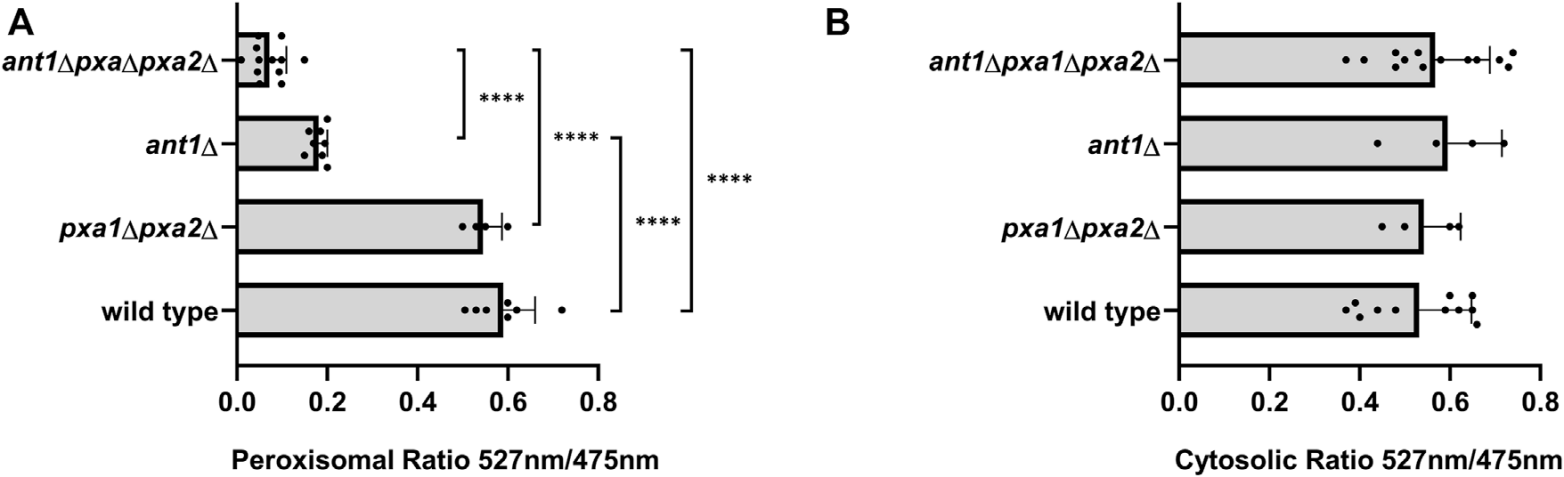
Comparison of ATP levels determined with FRET-based ATeam reporter proteins in wild-type and mutant yeast strains. Cells were transformed with the different ATeam reporter proteins, grown overnight in oleate medium and transferred to 25 mM MES buffer (pH 6.0) supplemented with 20 g/L glucose prior to measurements. **(A)** Peroxisomal ATP levels determined with reporter proteins ATeam-per and ATeam-per (mut) **(B)** ATP levels determined with reporter proteins ATeam-cyt and ATeam-cyt (mut). FRET was determined by measuring 527/475 nm. Ratios were calculated after subtraction of the background signal from wild-type cells transformed with an empty vector. The semi-relative ATP levels were then calculated by subtracting the 572/475 ratio of the ATeam(mut) reporter from the 572/475 of ATeam reporter. Data are means ± SD of values from 4–13 independent experiments. Only most relevant statistic relations are indicated in Figure 3, all are given in Supplementary Table S1. ****, ***, **, and * indicate significance with a *p*-value of *p* < 0.0001, *p* < 0.001, *p* < 0.01, and *p* < 0.05 respectively.

To provide *in vivo* evidence for the role of Ant1p and Pxa1p/Pxa2p in peroxisomal ATP uptake, we expressed modified versions of the FRET-based ATeam reporter protein (Imamura et al., 2009; Bermejo et al., 2010) in the different yeast strains to measure the relative ATP levels in the peroxisomes (ATeam-per), i.e. extended with a carboxy-terminal peroxisomal targeting signal) and the cytosol (ATeam-cyt). To correct for ATP-independent background fluorescence, we used mutated versions of the same reporter proteins that have no affinity for ATP (ATeam-per (mut) and ATeam-cyt (mut)). The relative ATP levels in cytosol and peroxisomes of *pxa1*Δ *pxa2*Δ mutant cells were similar as observed in cytosol and peroxisomes of wild-type cells (Figure 3). However, the relative ATP levels in peroxisomes of *ant1*Δ and *ant1*Δ *pxa1*Δ *pxa2*Δ mutant cells were significantly reduced (Figure 3A) while the relative cytosolic ATP levels in these strains were similar when compared to wild-type cells (Figure 3B). The observation that the relative peroxisomal ATP levels in the *ant1*Δ *pxa1*Δ *pxa2*Δ mutant cells were significantly lower than in the *ant1*Δ cells confirmed that Pxa1p/Pxa2p also mediates ATP uptake, in addition to Ant1p.

### Identification of an Additional Peroxisomal ATP Transporter

The ∼10% residual C8:0 β-oxidation activity measured in the *ant1*Δ *pxa1*Δ *pxa2*Δ mutant cells (Figure 1B) as well as the ∼20% residual *in vitro* ATP/ADP exchange observed in proteoliposomes prepared from peroxisomes of the *ant1*Δ *pxa1*Δ *pxa2*Δ mutant cells (Figure 2C), indicated the involvement of at least one additional peroxisomal ATP transporter. We hypothesized that in addition to Ant1p, which is a member of the MCF family and exclusively localized to peroxisomes, there might be one or more other members of this carrier family that have a dual localization in both the mitochondrial and the peroxisomal membrane. In order to study this, we developed a sensitive cell-based assay employing self-assembling GFP (Cabantous and Waldo, 2006) that allows to determine a possible peroxisomal localization of MCF proteins (see Supplementary Figure S2). To this end, we tagged the C-terminus of selected MCF proteins with the GFP(11) peptide sequence and co-expressed these with a peroxisome-targeted GFP(1–10)-PTS1 in wild-type yeast cells. We tested five mitochondrial MCF proteins that are known to function as adenine nucleotide carriers: Aac1p, Aac2p, Aac3p, Yea6p, and Leu5p. As positive control we used Ant1p, and as negative control we used the mitochondrial MCF protein Sco2p, which is known to mediate copper transport to cytochrome c oxidase and thus is assumed not to show co-localization in peroxisomes.

**FIGURE 4:**
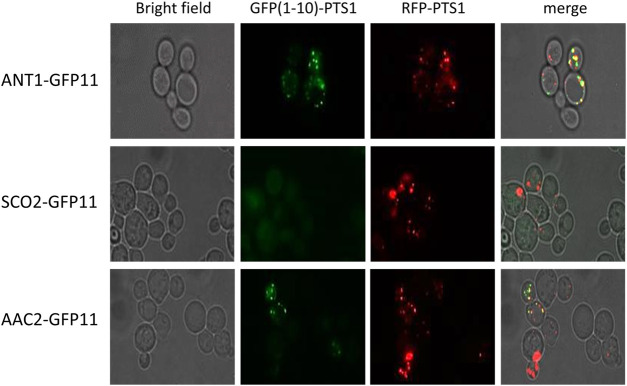
Peroxisomal localisation of Aac2p using self-assembling GFP assay. Wild-type cells were transformed with GFP(1–10)-PTS1, GFP(11)-tagged Ant1p, Sco2p, or Aac2p, as well as RFP-PTS1 for confirmation of peroxisomal localisation. All cells were cultured overnight in ethanol medium. Images show bright field to visualize the localization of the cells (left); fluorescence of self-assembling GFP (GFP(1–10)-PTS1; left centre), RFP-PTS1 (right centre) and the overlay of bright field, self-assembling GFP and RFP-PTS1 (Merge; right).

In contrast to GFP(11)-tagged Ant1p, we did not observe GFP fluorescence for GFP(11)-tagged Aac1p, Aac3p, Yea6p, Leu5p and Sco2p when co-expressed with peroxisome-targeted GFP(1–10)-PTS1. GFP(11)-tagged Aac2p, however, displayed a clear punctated GFP fluorescence pattern similar as observed for GFP(11)-tagged Ant1p, which indicated a peroxisomal co-localization. To confirm the peroxisomal co-localization of Aac2p, we co-expressed GFP(11)-tagged Aac2p with GFP(1–10)-PTS1 in wild-type cells expressing a peroxisomal RFP-PTS1 reporter protein. This revealed co-localization of the punctated green GFP fluorescence with the peroxisomal red RFP-PTS1 fluorescence similar as observed for Ant1p (Figure 4). These findings imply that Aac2p has a dual subcellular localization in both mitochondria and peroxisomes and thus could be responsible for the residual peroxisomal ATP in peroxisomes observed in the *ant1*Δ *pxa1*Δ *pxa2*Δ mutant cells. Interestingly, Aac2p was shown previously to function as an ATP/ADP carrier (Palmieri et al., 2006; Bamber et al., 2007; Klingenberg, 2008; Duncan et al., 2018), which fits very well with the residual *in vitro* ATP/ADP exchange we measured in proteoliposomes prepared from peroxisomes of the *ant1*Δ *pxa1*Δ *pxa2*Δ mutant cells (Figure 2C).

### Increased Mitochondrial Targeting of Aac2p Diminishes Residual Peroxisomal ATP Levels

**FIGURE 5:**
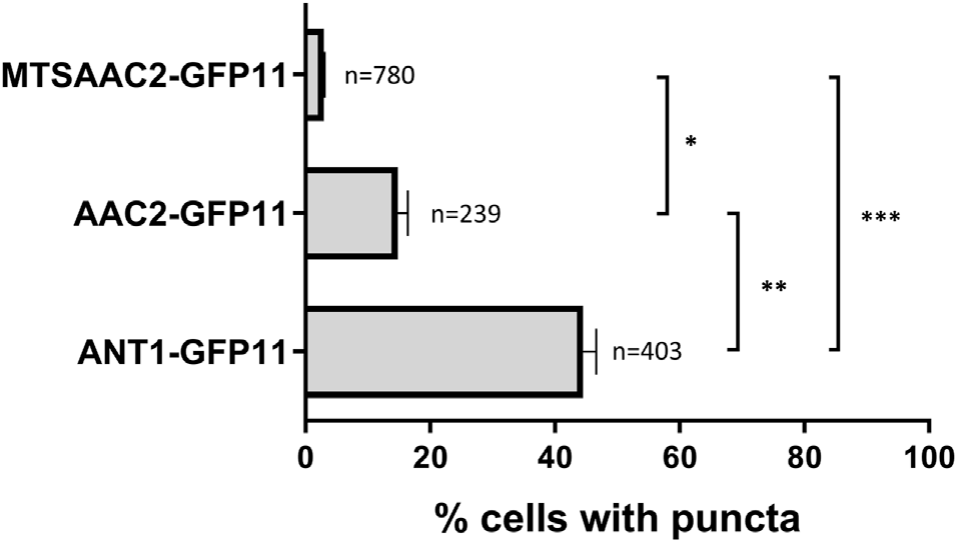
Quantification of cells with Ant1p, Aac2p or MTSAac2p located in the peroxisomal membrane. Wild-type cells co-expressing the indicated GFP(11)-tagged MCF protein and the peroxisomal GFP(1–10)-PTS1 protein were analyzed for the number of cells displaying punctated GFP fluorescence, resulting from the physical interaction between GFP(11)-tagged MCF proteins and the peroxisomal GFP(1–10)-PTS1 protein. Expressed as percentage of number of analyzed cells indicated next to the bars. A one-way ANOVA test with Tukey’s multiple comparisons test was performed. ***, **, and * indicate significance with a *p*-value of *p* < 0.001, *p* < 0.01, and *p* < 0.05 respectively.

In order to demonstrate that peroxisome-localized Aac2p is responsible for the residual C8:0 β-oxidation activity in the *ant1*Δ *pxa1*Δ *pxa2*Δ mutant cells, we attempted to delete the *AAC2* gene in these cells, but this did not result in a viable strain. As an alternative approach, we introduced a strong mitochondrial targeting signal (MTS) to the N terminus of Aac2p (van Roermund et al., 2016) in order to increase mitochondrial and decrease peroxisomal targeting of Aac2p. Using our self-assembling GFP assay, we observed that the addition of the strong MTS indeed reduced the peroxisomal localization of GFP(11)-tagged Aac2p to 2–3% of cells compared to 15% of GFP(11)-tagged Aac2p without the MTS (Figure 5).

**FIGURE 6:**
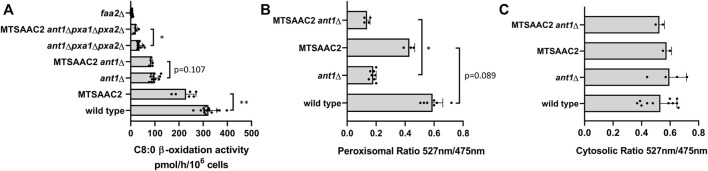
C8:0 β-oxidation activity and ATP levels in wild-type and mutant yeast strains expressing MTS-Aac2p. **(A)** Untransformed strains and the same strains expressing MTSAAC2 were cultured overnight in oleate medium and β-oxidation rates were measured using [1–^14^C] labelled octanoate (C8:0) as substrate. Data are means ± SD of values from 5–14 independent experiments. **(B)** Untransformed wild type and *ant1Δ* strains and the same strains expressing MTSAAC2 were transformed with ATeam-per or ATeam-per (mut) to determine relative peroxisomal ATP levels as described in legend of Figure 3. Data are means ± SD of values from 3–8 independent experiments. **(C)** Untransformed wild type and *ant1*Δ strains and the same strains expressing MTSAAC2p were transformed with ATeam-cyt and ATeam-cyt (mut) to determine relative cytosolic ATP levels as described in legend of Figure 3. Data are means ± SD of values from 2–11 independent experiments. Only most relevant statistic relations are indicated in Figure 6, all are given in Supplementary Table S1. ** and * indicate significance with a *p*-value of *p* < 0.01 and *p* < 0.05 respectively.

Next, we introduced the MTS sequence via homologous genomic recombination to the N terminus of Aac2p in wild-type, the *ant1Δ* and *ant1Δ pxa1Δ pxa2Δ* mutant cells. In all three strains, the expression of MTSAAC2 resulted in lower C8:0 β-oxidation activities when compared to the activities in the same strains that do not express MTSAAC2 (Figure 6A).

These findings strongly suggest that peroxisome-localized Aac2p indeed is responsible for the residual C8:0 β-oxidation activity observed in *ant1Δ pxa1Δ pxa2Δ* mutant cells.

We also observed a decrease in the peroxisomal ATP levels in wild-type cells expressing MTSAAC2, but this did not reach significance (*p* = 0.089) (Figure 6B). Thus, the combined activity of Ant1p and Pxa1p/Pxa2p appears sufficient to maintain the ATP levels in wild-type cells expressing MTSAAC2. Expression of MTSAAC2 in *ant1Δ* cells resulted in a significant decrease in ATP levels. We did not study the effect of MTSAAC2 on the peroxisomal ATP levels in *ant1Δ pxa1Δ pxa2Δ* mutant cells because the residual ATP levels in this mutant strain were already near the detection limit of the ATeam reporter proteins.

### Human ABCD1, ABCD2, and ABCD3 Can Also Transport ATP

After having established that Pxa1p/Pxa2p can mediate ATP uptake into peroxisomes, we studied whether the human orthologues HsABCD1, HsABCD2, and HsABCD3 can also mediate peroxisomal ATP uptake in addition to transport of fatty acyl-CoAs. We previously showed that yeast-codon optimized HsABCD1, HsABCD2 and HsABCD3 can be functionally expressed as homodimers in *S. cerevisiae*, and display different substrate specificities (van Roermund et al., 2014).

**FIGURE 7:**
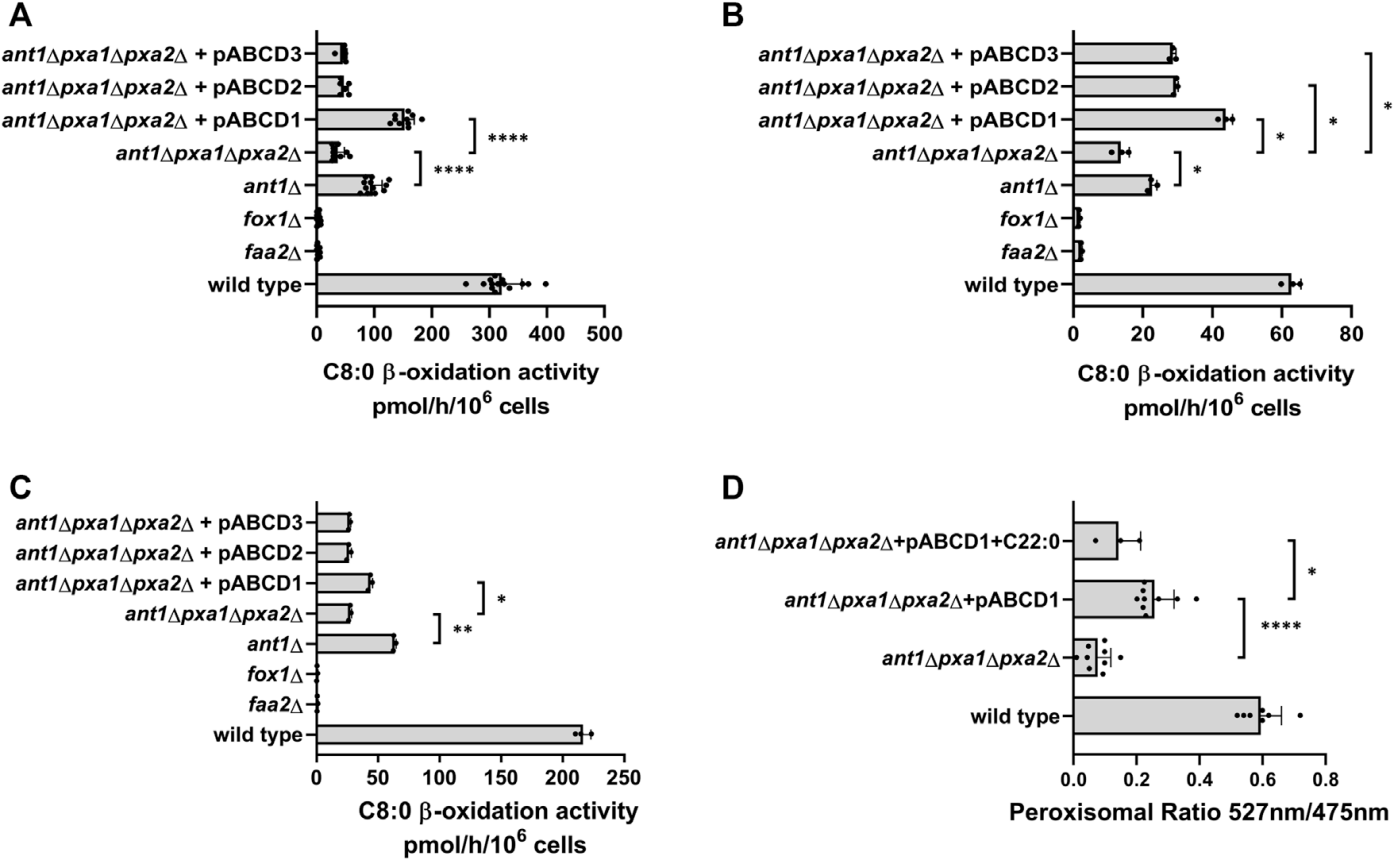
C8:0 β-oxidation activity in wild-type and mutant yeast strains expressing human ABCD proteins. Wild-type and mutant strains, including *ant1Δpxa1Δpxa2Δ* strains expressing ABCD1, ABCD2, or ABCD3 were cultured overnight in oleate medium **(A)**, ethanol medium **(B)** or oleate medium supplemented with 100 µM C22:0 **(C)**. Fatty acid ß-oxidation activity was measured with [1–^14^C] labelled octanoate (C8:0) **(A–C)**. **(D)** Wild-type and mutant strains, including *ant1Δpxa1Δpxa2Δ* strains expressing ABCD1 were transformed with ATeam-per and ATeam-per (mut) to allow quantification of peroxisomal ATP levels as described in legend of Figure 3. Cells were grown overnight in oleate medium or oleate medium supplemented with 100 µM C22:0. Data are means ± SD of values from 3–12 independent experiments. Only most relevant statistic relations are indicated in Figure 7, all are given in Supplementary Table S1. ****, ***, **, and * indicate significance with a *p*-value of respectively *p* < 0.0001, *p* < 0.001, *p* < 0.01, and *p* < 0.05.

Expression of HsABCD1 in *ant1*Δ *pxa1*Δ *pxa2*Δ mutant cells cultured on oleate medium resulted in a more than 3-fold increase in C8:0 β-oxidation activity (Figure 7A) and co-expression with the peroxisomal ATeam reporter proteins showed a marked increase in the relative peroxisomal ATP levels (Figure 7D). Thus, similar as its yeast orthologues, HsABCD1 can also mediate ATP uptake. Expression of ABCD2 or ABCD3 in *ant1*Δ *pxa1*Δ *pxa2*Δ mutant cells did not result in a significant increase in C8:0 β-oxidation activity (Figure 7A).

C8:0 β-oxidation activity was not increased when HsABCD2 or HsABCD3 were expressed in *ant1*Δ *pxa1*Δ *pxa2*Δ mutant cells cultured on oleate medium (Figure 7A). Since the CoA ester of oleate, oleoyl-CoA, is also a good substrate for HsABCD2 and HsABCD3, but less for HsABCD1, we hypothesized that the uptake of oleoyl-CoA competes for the uptake of ATP by the ABCD proteins. To study this, we cultured the *ant1Δ pxa1Δ pxa2Δ* cells expressing HsABCD1, HsABCD2, or HsABCD3 on ethanol instead of oleate and repeated the C8:0 β-oxidation activity measurements. Under these conditions we indeed measured not only a significant increase in C8:0 β-oxidation activity in the cells expressing HsABCD1 but also in the cells expressing HsABCD2 or HsABCD3 when compared to the same cells expressing neither of these proteins (Figure 7B).

The substrate preference of ABCD1 for very-long-chain acyl-CoAs also allowed us to study whether acyl-CoA and ATP compete for the same binding site of ABCD1. To this end, we measured C8:0 β-oxidation activity in oleate-grown cells in the presence (Figure 7C) and absence (Figure 7A) of docosanoic acid (C22:0), which is a good substrate for ABCD1 after intracellular conversion into its CoA-ester (van Roermund et al., 2014). Both the C8:0 β-oxidation activity (Figure 7C) and the peroxisomal ATP levels (Figure 7D) were lower in the presence of C22:0, which strongly suggests that the fatty acyl-CoAs and ATP compete for the same binding site in HsABCD1.

Taken together, these results show that all three human ABCD transporters can mediate peroxisomal ATP uptake in addition to transport of fatty acyl-CoAs.

## Discussion

Peroxisomes are generally considered to be selectively permeable organelles, which allow molecules with an Mw < 400 Da to cross the peroxisomal membrane passively via one or more (putative) channel-forming proteins, including Pxmp2 (Rokka et al., 2009) and Pex11beta (van Roermund et al., 2000; Mindthoff et al., 2016). Transport across the peroxisomal membrane of more bulky molecules, such as fatty acyl-CoAs and ATP, however, requires dedicated transport proteins. Among others, intra-peroxisomal ATP is essential for the peroxisomal β-oxidation of fatty acids. Previous work has shown that the transport of fatty acyl-CoAs is mediated by different dimeric half-ABC transporters of the ABCD family, which includes the heterodimer Pxa1p/Pxa2p in peroxisomes of *S. cerevisiae* and homodimers of ABCD1, ABCD2, and ABCD3 in human peroxisomes. In this paper we have studied which proteins are involved in the peroxisomal uptake of ATP. ATP has to be transported from the cytosol into the peroxisome since peroxisomes do not possess an ATP-synthesizing or regenerating system. To this end, we generated and used a series of *S. cerevisiae* mutant strains in which we deleted single or combinations of genes encoding putative peroxisomal ATP transporters. As readouts for peroxisomal ATP uptake we used three different and independent assays, including 1) measurement of *in vivo* C8:0 β-oxidation activity, which in yeast is strictly dependent on intra-peroxisomal ATP; 2) *in vitro* ATP uptake/exchange by proteoliposomes prepared from peroxisomal membranes isolated from the different mutant strains; and 3) *in vivo* measurement of relative ATP levels using FRET-based ATeam reporter proteins targeted to the peroxisomes or the cytosol.

We demonstrated that peroxisomes in *S. cerevisiae* contain at least three different transport systems that can mediate ATP uptake, each with a different mode of action. As anticipated, we found the previously reported peroxisomal membrane protein Ant1p to be responsible for most of the peroxisomal ATP uptake as can be concluded from the observation that *in vivo*, a deletion of *ANT1* resulted in a ∼70% reduction of the peroxisomal ATP-dependent C8:0 β-oxidation activity and in a marked reduction of the intra-peroxisomal ATP levels. As reported previously and confirmed in our *in vitro* proteoliposome uptake studies, Ant1p functions as an antiporter of ATP against AMP and ADP (Palmieri et al., 2001; van Roermund et al., 2001). Unexpectedly, we found that the remaining peroxisomal ATP uptake capacity is mediated by the ABC transporter protein complex Pxa1p/Pxa2p, which most probably functions as an ATP uniporter, and Aac2p, a predominantly mitochondrial ATP/ADP antiporter (Bamber et al., 2007; Klingenberg, 2008; Duncan et al., 2018; van Roermund CWT et al., 2020), which we here showed to be partly localized in peroxisomes.

Our finding that heterodimeric Pxa1p/Pxa2p, as well as the human orthologues HsABCD1, HsABCD2, and HsABCD3 as homodimers, can also mediate peroxisomal uptake of non-hydrolyzed ATP in addition to fatty acyl-CoAs was unexpected and is very intriguing. Indeed, ABC transporter proteins typically use ATP hydrolysis to catalyze the transport of substrates across membranes, although few ABC transporter proteins have been reported to function as ATP channels (Abraham et al., 1993; Reisin et al., 1994; Roman et al., 1997; Jansen et al., 2014). Our *in vivo* substrate competition experiments in which we added C22:0 during the C8:0 β-oxidation measurement suggested that ATP and acyl-CoAs compete for the same binding site of HsABCD1, which thus is different from the binding site at which ATP is hydrolyzed to drive the transport of fatty acyl-CoAs across the peroxisomal membrane. This competition is also suggested by the significant increase in C8:0 β-oxidation activity when the *ant1*Δ *pxa1*Δ *pxa2*Δ cells expressing HsABCD1, HsABCD2, or HsABCD3 are cultured on ethanol instead of oleate. We recently reported that Pxa1p/Pxa2p is also involved in peroxisomal uptake of CoA (Roermund et al., 2021). This may suggest that the increased C8:0 β-oxidation observed in the *ant1*Δ *pxa1*Δ *pxa2*Δ cells expressing HsABCD1 and cultured on ethanol could also be due to an ABCD1-mediated increase of intra-peroxisomal CoA levels. However, this explanation seems unlikely because if intra-peroxisomal CoA would be limiting for C8:0 β-oxidation, one would expect that addition of C22:0, which is a good substrate for ABCD1, would increase intra-peroxisomal CoA levels resulting in increased C8:0 βoxidation, while we observed a decrease of the latter. Unfortunately, we and others have not succeeded in reconstituting purified ABCD proteins in liposomes to study these and other aspects in more detail *in vitro*.

Aac2p is a well-established ATP/ADP antiporter localized in the inner mitochondrial membrane (Bamber et al., 2007; Klingenberg, 2008; Duncan et al., 2018; van Roermund CWT et al., 2020). Our finding that Aac2p is partly localized in peroxisomes and thus constitutes a third protein mediating peroxisomal ATP uptake is equally intriguing as the finding of the involvement of Pxa1p/Pxa2p. It also raises the question on how Aac2p is targeted to peroxisomes in addition to its primary targeting to mitochondria, given that the mechanisms of membrane protein import into mitochondria and peroxisomes must be very different, although this is still largely unknown for peroxisomal membrane proteins. It should be noted, however, that a dual localization in mitochondria and peroxisomes is not unique and has been reported for several proteins, including DLP1, FIS1, MIRO, and VDAC (Costello et al., 2018). In the case of Aac2p, the introduction of a stronger mitochondrial targeting signal (MTS) to its N terminus reduced the peroxisomal localization and, consequently, reduced the peroxisomal ATP uptake mediated by peroxisomal Aac2p.

The different substrate affinities and modes of action of Ant1p, Pxa1p/Pxa2p, and Aac2p not only ensure that peroxisomes can maintain their ATP levels to support the intra-peroxisomal ATP-dependent enzyme reactions, but also take care of the export of AMP and ADP generated after intra-peroxisomal hydrolysis of ATP. Indeed, peroxisomes in yeast harbor several enzymes the activity of which depends on ATP hydrolysis leading to the generation of AMP or ADP. These include several acyl-CoA synthetases (Hettema et al., 1996; van Roermund et al., 2012), PCD1 (Cartwright et al., 2000), NPY1 (Xu et al., 2000), VPS34 kinase (Stjepanovic et al., 2017) and LonP proteases (Bartoszewska et al., 2012; Pomatto et al., 2017).

Taken together, our results provide compelling evidence for the presence of multiple systems for the uptake and exchange of ATP in peroxisomes in yeast. Our finding that also the human peroxisomal ABC transporters can mediate peroxisomal ATP uptake strongly suggests that our findings in yeast are transferable to humans.

## Methods

### Yeast Strains

We used *S. cerevisiae* BJ1991 (*MATα, pep4-3, prbl-1122, ura3-52, leu2, trp1*) as wild-type strain and for the generation of targeted deletion mutant strains. Gene deletions in BJ1991 were created by replacement of specific genes by the yeast *LEU2* gene, the Kanamycin (*KAN*) or the Bleomycin (*BLE*) resistance gene using homologous recombination. For this study we generated and used the following deletion mutant strains: *ant1Δ* (*ant1*: *KAN*), *pxa1Δ pxa2Δ* (*pxa1*:*LEU2*, *pxa2*:*KAN*), and *ant1Δ pxa1Δ pxa2Δ* (*ant1*:*KAN*, *pxa1*:*LEU2*, *pxa2*:*BLE*), and used two previously described mutant strains *faa2Δ* (*faa2*:*LEU2*) and *fox1Δ* (*fox1*:*KAN*).

### Culture Conditions

We cultured yeast cells at 28°C under continuous shaking at 225 rpm. For standard growth, cells were cultured in glucose medium containing 6.7 g/L yeast nitrogen base without amino acids (Difco) and 5 g/L D-glucose. Amino acids were supplemented to the medium when required; 30 mg/L leucine, 20 mg/L uracil, or 20 mg/L tryptophan. To induce peroxisome proliferation, yeast cells were cultured for at least 24 h in glucose medium and then transferred to and cultured overnight in YPO medium (3 g/L yeast extract, 5 g/L peptone, 25 mM potassium phosphate buffer (pH = 6), 1.07 g/L oleate, 2.16 g/L Tween-80) with supplemented amino acids when required.

For fluorescent microscopy of self-assembling GFP, yeast cells were cultured in ethanol medium containing 1 g/L yeast extract, 25 mM potassium phosphate buffer (pH = 6), 6.7 g/L yeast nitrogen base and 2% ethanol and supplemented amino acids when required.

### Octanoate (C8:0) β-Oxidation Measurements

We measured β-oxidation activity in intact yeast cells as follows. Cells were cultured overnight in YPO media, harvested by centrifugation, washed and resuspended in 9 g/L NaCl at a cell density of OD_600 nm_ = 1 (∼1.48 × 10^7^ cells/mL). Incubations were performed in 20 mL vials with a rubber septum, containing two tubes, one with the cells in incubation mixture and the other with 500 μL NaOH (2 M). To start the measurements, 20 μL of cell suspension was added to the reaction mixture composed of 20 μL MES buffer (0.5 M; pH = 6), 140 μL NaCl (9 g/L), and 20 μL of 100 μM [1–^14^C] octanoate (200,000 dpm) as substrate. The reaction was allowed to proceed for 1 h at 28°C after which the reaction was terminated by the addition of 50 μL of perchloric acid (2.6 M). Radiolabelled [^14^C]-CO_2_, released during the β-oxidation of octanoate was trapped overnight in the tube with 500 µL of 2 M NaOH. Acid soluble products (ASP) were collected after extraction with chloroform/methanol/heptane (van Roermund CWT et al., 2020). Both CO_2_ and ASP were quantified in a liquid scintillation counter and the β-oxidation rate was determined as the sum of CO_2_ and ASP production. The octanoate β-oxidation rate in wild-type cells was 3.2 ± 0.4 nmol/h/10^7^ cells.

### ATP Uptake Measurements in Proteoliposomes

We isolated peroxisomes in duplicate from wild-type and the *ant1Δ*, *pxa1Δpxa2Δ* and *ant1Δpxa1Δpxa2Δ* mutant strains cultured overnight in oleate medium using cell fractionation and Nycodenz gradient centrifugation as described previously (van Roermund et al., 2001). Gradient fractions were analysed for peroxisomal 3-hydroxyacyl-CoA dehydrogenase (3-HAD) and mitochondrial fumarase activity (van Roermund et al., 2001). Purified peroxisomes from fractions 2–4 of the gradients (Supplementary Figure S1) and equivalent to 375 units of peroxisomal 3HAD activity were harvested and dissolved in 150 µL of 50 mM Hepes (pH = 7.4) and 5 mM MgCl_2_. Of these, peroxisomes equivalent to 50 units of 3HAD activity were added to 1 mL 30 g/L L-α-glycerophosphorylcholine only or supplemented with 10 mM ADP or 10 mM AMP after which the mixtures were frozen in liquid nitrogen. The samples were then thawed at room temperature, resulting in the formation of proteoliposomes, and subjected to size-exclusion chromatography using Sephadex G-25 (Medium) columns (GE Healthcare Life Science) to remove external ADP or AMP. The eluate was used to start the uptake experiment by adding 0.2 mM [α-^32^P]-ATP (6,000 Ci/mmol). The uptake reaction was terminated via passing the proteoliposomes over Dowex AG1-X8 anion-exchange columns using 150 mM sodium acetate (pH = 7.4) as elution buffer. The incorporated [α-^32^P]-ATP was quantified by liquid scintillation counting. Time-dependent uptake data were fitted using nonlinear regression analysis based on one-phase exponential association using GraphPad Prism 5.0 software (GraphPad, www.graphpad.com). The initial velocity of uptakes were calculated using the equation slope = (Plateau–Y0)*k, with Y0 set to 0.

### Measurement of ATP Levels Using FRET-Based ATeam Reporter Proteins

We measured the relative *in vivo* ATP levels in peroxisomes and the cytosol of wild-type cells and different mutant strains through expression of modified versions of the previously described ATeam sensors (Imamura et al., 2009). As source for the generation of the ATeam reporter proteins used in this study, we ordered the pDR-GW AT1.03 and pDR-GW AT1.03 R122K/R126K plasmids (Bermejo et al., 2010) from Addgene (deposited by Wolf Frommer). The pDR-GW AT1.03 plasmid (http://www.addgene.org/28003) codes for a cytosolic ATeam reporter protein and the pDR-GW AT1.03 R122K/R126K plasmid (http://www.addgene.org/28005) codes for a mutated version of the same ATeam reporter protein that no longer binds ATP. To allow constitutive, carbon source-independent ATeam gene expression in yeast, we first replaced the CTA1 promoter of pIJL30 with the TEF1 promoter generating the yeast expression vector pMK05 (TEF1pr, ARS1/CEN4, Trp1, ampR). The *XbaI*-*HindIII* fragments from pDR-GW AT1.03 and pDR-GW AT1.03R122K/R126K were then subcloned downstream of the TEF1 promoter into the *XbaI*-*HindIII* sites of pMK05. The resulting plasmids were designated pATeam-cyt (expressing cytosolic AT1.03) and pATeam-cyt (mut) (expressing the mutated cytosolic AT1.03 R122K/R126K), respectively.

To target the ATeam reporter proteins to peroxisomes, we replaced by site directed mutagenesis the stop codon of the AT1.03 ORFs in pATeam-cyt and pATeam-cyt (mut) by a flexible loop and the coding sequence for the twelve C-terminal amino acids of Fox2p, including the strong peroxisomal targeting sequence PTS1. This resulted in pATeam-per (expressing peroxisomal AT1.03) and pATeam-per (mut) (expressing the mutated peroxisomal AT1.03 R122K/R126K), respectively.

Yeast cells were transformed with the different pATeam plasmids, plated and individual colonies were cultured overnight in YPO medium. The cells were harvested by centrifugation at 230 *g* for 5 min at 4°C, washed once with and then suspended in cold MES-glucose buffer (pH 6.0) composed of 25 mM 2-(N-morpholino) ethanesulfonic acid and 20 g/L D-glucose. We resuspended the cells in MES + glucose to assure that the cytosolic ATP levels (i.e. the substrate for import) during the measurement were comparable in all strains (as confirmed in Figures 3B, 6C) and thus would not provide an additional variable in the measurement of peroxisomal ATP import. Cells were kept on ice until analysis was conducted (5 h max). Prior to the measurements, the suspended cells were diluted with MES-glucose buffer until OD_600 nm_ = 3, and 200 μL was transferred in duplicate into a 96-wells microplate (Greiner, black round bottom). FRET analysis was then performed in on a Tecan Infinite M200 pro plate reader using an excitation of 435/9 nm and detecting emission at 475/20 nm and 527/20 nm, respectively. Fluorescence intensities at each wavelength were measured 10 times. To assure accurate measurements, prior to the start of each cycle the microplate was shaken in orbitals with an amplitude of 2 mm, a Z position height of 20,000 μm, settle time of 200 ms, and 0 s lag time. Ratios were calculated after subtraction of the background signal in wild-type cells transformed with an empty plasmid (no ATeam expression). The relative ATP levels were obtained by subtracting the 572/475 ratio of the ATeam-cyt/per (mut) reporter protein from the 572/475 ratio of the corresponding ATeam-cyt/per reporter protein.

### Construction of ABCD Expression Plasmids

We designed and ordered a yeast-codon optimized open reading frame (ORF) coding for ABCD1 and flanked by *SacI* and *KpnI*, and cloned this into the yeast expression vectors pIJL30 and pEL30. Construction of ABCD2 and ABCD3 expression plasmids have been described previously (van Roermund et al., 2014).

### Subcellular Localisation of MCF Proteins Using a Self-Assembling GFP Assay

We adapted the self-assembling GFP assay (Cabantous and Waldo, 2006) to study the subcellular localization of MCF proteins. To this end, we designed and ordered from Genscript a yeast-codon optimized open reading frame (ORF) coding for GFP(1–10) in pUC57 and re-cloned this into the yeast expression vector pIJL30 (CTA1pr, ARS1/CEN4, Trp1, ampR) allowing cytosolic expression of GFP(1–10). To generate a peroxisome-localized GFP(1–10), we used the pUC57-GFP(1–10) as template and added via PCR amplification the coding sequences of the twelve C-terminal amino acids of FOX2, which include a strong peroxisomal targeting signal PTS1, spaced with a flexible linker. The resulting GFP(1–10)-PTS1 ORF was also subcloned into the pIJL30 expression vector.

To generate a basic cloning vector that allows expression of MCF proteins with a C-terminal extension coding for the GFP(11) domain, we introduced a linker encoding a flexible loop and yeast-codon optimized GFP(11) into the *BamH1* and *HindIII* sites of yeast expression vector pEL30 (van Roermund et al., 2012) (CTA1pr, ARS1/CEN4, URA3, ampR). The resulting plasmid pEL30-GFP(11) allows upstream cloning of ORFs into *SacI*, *KpnI*, *SmaI* and *BamHI* sites in frame with GFP(11).

ORFs encoding the yeast MCF proteins Ant1p, Aac1p, Aac2p, Aac3p, Yea6p, Leu5p, and Sco2p were PCR amplified from genomic DNA of *S. cerevisiae* using ORF-specific PCR primers with small extensions to introduce the appropriate restriction sites and, after restriction, sub-cloned in frame with GFP(11) into pEL30-GFP(11). All PCR-amplified sequences were verified by Sanger sequencing.

Wild-type cells were co-transformed with pEL30-GFP(11) containing one of the MCF proteins and either pIJL30-GFP(1–10)-PTS1 or pIJL30-GFP(1–10). After transformation the cells were cultured in 2% ethanol medium for 24 h, harvested, re-suspended in sterile water and examined on a ZEISS Axio Observer A1 fluorescence microscope using a 450 nm excitation and a 515–565 nm emission filter. The Leica Application Suite was used to capture the images.

### Enhancing Mitochondrial Targeting of Aac2p

We introduced the mitochondrial targeting signal (MTS) from the mitochondrial succinate/fumarate carrier of *Arabidopsis thaliana* to the N-terminus of Aac2p (van Roermund et al., 2016), to increase mitochondrial and decrease peroxisomal targeting of Aac2p. To this end, we amplified the ORF of *AAC2* by PCR using an *AAC2*-specific forward primer with a 5′ extension comprising the coding sequence for the MTS. The MTS-*AAC2* ORF was cloned in frame with GFP(11) into pEL30-GFP(11) and verified by Sanger sequencing. Wild-type cells were co-transformed with the resulting pEL30-MTS-Aac2p-GFP(11) vector and the pIJL30-GFP(1–10)-PTS1 vector and used in the self-assembling GFP assay described above to compare the subcellular localization of MTS-Aac2p with Aac2p.

In order to introduce the MTS by homologous recombination at the N terminus of genomically encoded Aac2p, we generated by PCR amplification a DNA fragment comprising a 5′ *AAC2* non-coding sequence followed by the *NAT1* resistance gene under control of the *TEF1* promoter, the sequence for the MTS under control of the *NOP1* promoter, and a 5′ *AAC2* coding sequence. The fragment was transformed into wild-type, *ant1Δ* and *ant1/pxa1/pxa2Δ* mutant strains. After transformation, cells were washed and incubated for 5 h in 5 g/L glucose supplemented with amino acids, so that the *NAT1* resistance gene could be expressed. Cells were then plated on YPD plates supplemented with 100 μg/mL NTC to select for cells expressing the *NAT1* gene. Correct integration of the DNA fragment at the *AAC2* locus was verified by Sanger sequencing. Normal growth was observed in all knock-in strains on either YPD or 5 g/L glucose medium supplemented with amino acids. The different knock-in strains were used to determine the relative peroxisomal and cytosolic ATP levels using the ATeam reporter constructs as described above.

## Data Availability

The original contributions presented in the study are included in the article/Supplementary Material, further inquiries can be directed to the corresponding author.
