# Supplementary figures and images for: Peroxisomal ATP Uptake Is Provided by Two Adenine Nucleotide Transporters and the ABCD Transporters
Source: Front Cell Dev Biol. 2022 Jan 19;9:788921. doi: 10.3389/fcell.2021.788921 (PMC8807639; doi:10.3389/fcell.2021.788921)

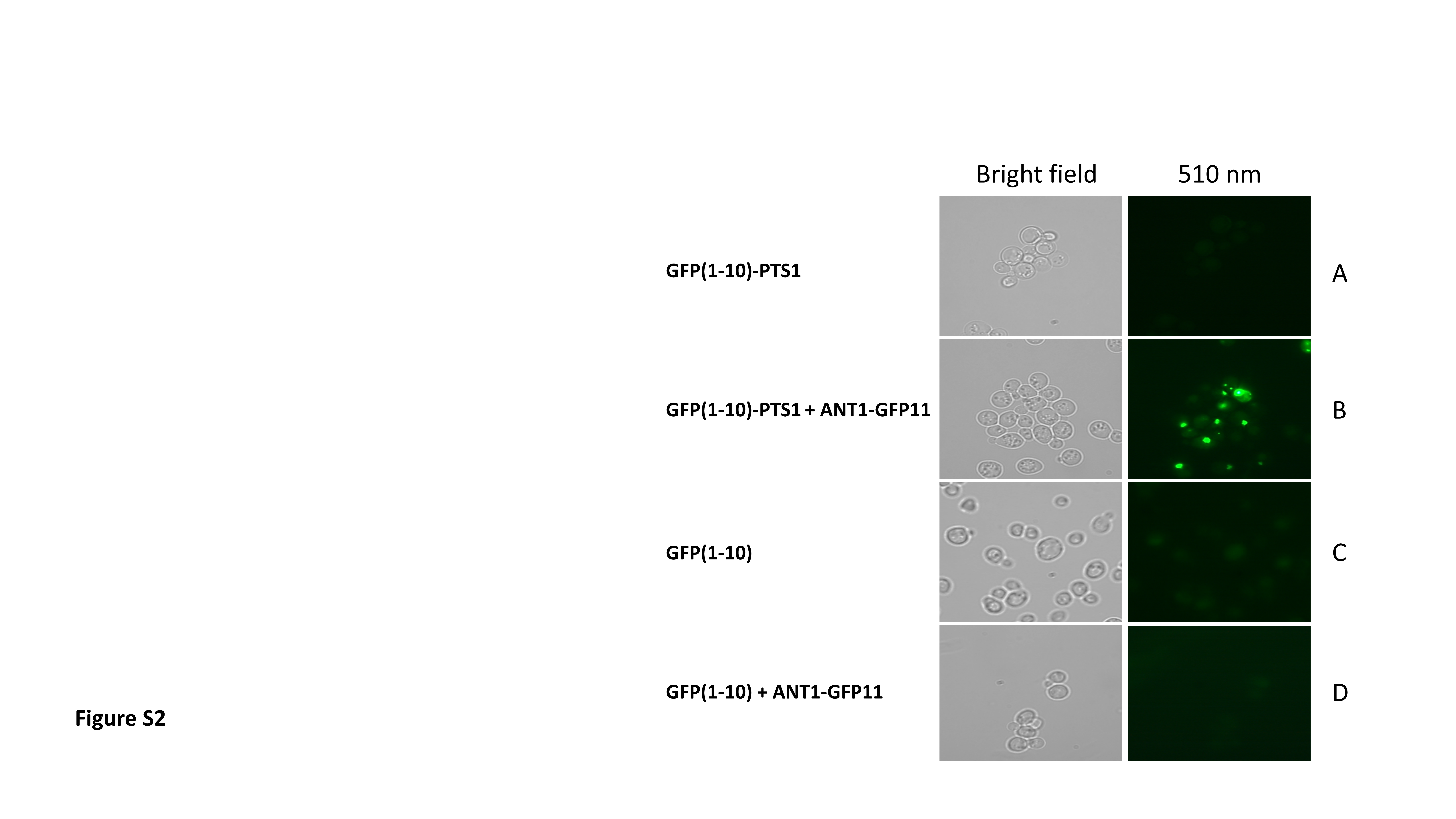

Supplement: Supplementary file 1 [file Image2.tif]

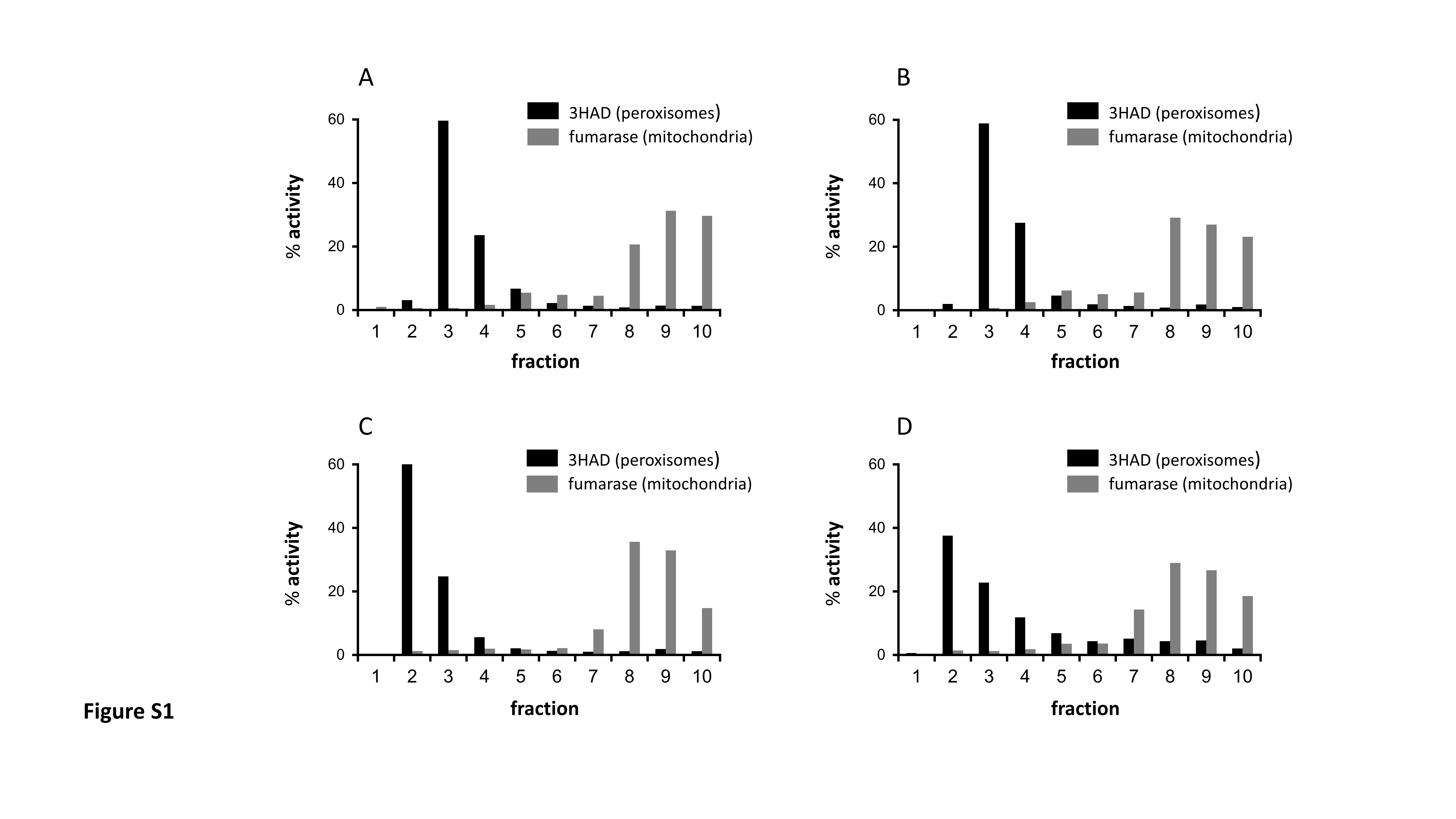

Supplement: Supplementary file 2 [file Image1.tif]
